# High Expression of IL-36γ in Influenza Patients Regulates Interferon Signaling Pathway and Causes Programmed Cell Death During Influenza Virus Infection

**DOI:** 10.3389/fimmu.2020.552606

**Published:** 2020-10-22

**Authors:** Shuai Liu, Hui Li, Yeming Wang, Haibo Li, Sisi Du, Xiaohui Zou, Xulong Zhang, Bin Cao

**Affiliations:** ^1^ China-Japan Friendship Hospital, National Clinical Research Center for Respiratory Diseases, Clinical Center for Pulmonary Infections, Capital Medical University, Beijing, China; ^2^ Department of Pulmonary and Critical Care Medicine, Center for Respiratory Diseases, China-Japan Friendship Hospital, Beijing, China; ^3^ Institute of Respiratory Medicine, Chinese Academy of Medical Sciences, Peking Union Medical College, Beijing, China; ^4^ Department of Immunology, School of Basic Medical Sciences, Capital Medical University, Beijing, China; ^5^ Tsinghua University-Peking University Joint Center for Life Sciences, Tsinghua University, Beijing, China

**Keywords:** IL-36, influenza infection, interferons signaling pathway, programmed cell death, acute respiratory distress syndrome

## Abstract

As a severe complication of influenza infection, acute respiratory distress syndrome (ARDS) has higher morbidity and mortality. Although IL-36γ has been proven to promote inflammation at epithelial sites and protect against specific pathogen infection, the detailed roles in severe influenza infection remain poorly understood. In this study, we have found that the expression of IL-36γ is higher in influenza-induced ARDS patients than healthy individuals. IL-36γ was induced in human lung epithelial cells and peripheral blood mononuclear cells by Influenza A virus (IAV) infection, and its induction was synergistically correlated with initiation of the cyclooxygenase-2 (COX-2)/Prostaglandin E2 (PGE2) axis. We also have found that expression of superficial IL-36R was elevated in severe influenza patients and in IAV-stimulated cells. Furthermore, although IL-36γ enhanced the induction of type I and III interferons (IFNs), which promoted IAV-mediated IFN-stimulated STAT1 and STAT2 phosphorylated inhibition in lung epithelial cells, the downstream interferon-stimulated genes (ISGs) were not affected. Finally, we have revealed that IL-36γ treatment could promote apoptosis and inhibit autophagy in the early stages of IAV infection. Overall, these findings demonstrated IL-36γ is a critical host immune factor in response to IAV infection. It has potential activity in the regulation of the interferon signaling pathway and was involved in different types of programmed cell death in human airway epithelial cells as well.

## Introduction

Despite the widespread use of antiviral drugs and broad availability of vaccines, influenza still remains a burden to public health and has the potential to cause a global pandemic in humans (1). Some special populations, particularly children or the elderly, tend to progress into refractory hypoxemia and even acute respiratory distress syndrome after influenza virus infection (2). Thus, a detailed understanding of pathogenesis and immunological determinants of severe influenza infection benefits clinical treatment of these patients.

The lung is the main target organ of influenza, and acute lung injury induced by influenza infection is an important reason for adverse clinical outcomes. The severity of acute lung injury is often associated with degree of viruses-stimulating host immune response, virus replication, and virus-induced cell death (3). Many inflammatory cytokines, chemokines, and immunoregulatory cytokines induced by influenza infection can interact with host proteins to affect viral replication, immune regulation, and induce programmed death. Influenza viruses could activate respiratory epithelium *via* the extracellular or intracellular TLRs, RIG-1, and NLRP3 inflammasome, causing a large amount of immunoregulatory cytokines and antiviral factors release, such as type I and III interferons (IFNs), IL-1 family members, IL-12 family members, tumor necrosis factor (TNF)-α, and macrophage inflammatory protein (MIP)-α/β (4–8). However, there is still limited knowledge about cytokines, which is induced by influenza infection and its function as regulator and mediator in host-virus interaction.

IL-36, a cytokine recently described as a member of larger IL-1 family, including three agonist proteins (IL-36α, IL-36β, and IL-36γ) and antagonist IL-36Ra, are produced in stimulated epithelial cells and a variety of immune cells, such as monocytes, macrophages, and dendritic cells (9). IL-36 utilize the heterodimeric IL-36 receptor (IL-36R) and IL-1 receptor accessory chain (IL-1RAcp) for activation of downstream inflammatory signaling pathways and acts as proinflammatory cytokines (10). Many studies suggest IL-36 cytokines play a vital role in lung disorders, especially lung infection and secondary inflammatory response, but with contradictory results. On the one hand, IL-36γ secreted *via* packaging within microparticles and played a vital proximal role in lung innate mucosal immunity during bacterial pneumonia *via* induction of type-I cytokine responses and polarization of classical macrophage (11); on the other hand, IL-36γ derived from alveolar epithelial cells and pulmonary macrophages during *Pseudomonas aeruginosa* infection yet contributes to deleterious effects on host immune response (12). Likewise, some results regarding the role of IL-36 in influenza infection and pathogenesis *in vivo* remain a matter of debate. One study shows that IL-36 deficient mice can protect against influenza virus-induced lung damage and mortality by limiting lung inflammation (13). Another study reports a protective role of IL-36 *in vivo* during influenza infection *via* promoting lung alveolar macrophages survival and limiting viral replication (14). This observation raises the possibility that IL-36 induction plays a significant role in lung pathologic conditions, especially in lung infection and pulmonary inflammation. Therefore, a more thorough understanding of the function of IL-36 in severe influenza patients may provide appropriate intervention leading to better inflammation and viral control.

Programmed cell death (PCD) plays a vital role in balancing cell death and survival of normal cells, but this homeostasis could be disturbed when cells are infected with influenza virus or sense excessive stresses. Of note, apoptosis and autophagy are the main forms of PCD, which can be easily distinguished by their morphological characteristics (15). One transcriptome study highlights that apoptosis related genes are induced and expressed at the early stage after influenza virus infection, which can be considered primarily as a cellular response mechanism to fight the invading pathogen and limit spread of infection (16). Modulation of autophagic flux as well as induction of intracellular oxidative stress also happens during IAV infection. Several studies have reported that influenza virus infection can promote the formation of autophagosomes in the cytoplasm, and then facilitate self-replication by inhibiting the degradation of autolysosomes, which easily increase virus-induced cell damage (17). This whole process of PCD induced by influenza virus infection can be accomplished mainly by the synergistic action of various cytokines and viral proteins. However, many cellular factors, such as newly discovered cytokines and related pathway in this process, have not been fully investigated.

Our study aimed to better understand the role of IL-36 in influenza infection, especially in severe influenza patients and their vulnerable respiratory epithelial structure. Herein, we describe the characteristics of IL-36 family members expressed in influenza-induced ARDS patients and present the induction and related mechanisms of IL-36α/γ in airway epithelial cells after influenza infection. We identify the extent to which IL-36γ impacts IAV infection *via* regulating interferon signaling pathway and causing programmed cell death during infection.

## Materials and Methods

### Ethics Statement

Experiments involving human participants were conducted according to the declaration of Helsinki and approved by the China-Japan Friendship Hospital Ethics Committee (Approval No. 2018-120-K86) in accordance with its guidelines for the protection of human subjects. The participants provided their written informed consent to participate in this study.

### Clinical Samples

Peripheral blood samples were obtained from 20 patients with influenza-induced ARDS who were admitted to China-Japan friendship hospital. We identified patients with influenza-induced ARDS who confirmed influenza (influenza A or B) virus positive *via* nucleic acid detecting from sputa, throat swabs, or bronchoalveolar lavage fluid (BALF) samples. Simultaneously, lung injury was evaluated for the ARDS patients with influenza viral pneumonia. All the patients involved have no other putative respiratory viruses identified. Clinical characteristics of patients enrolled in this study appear in **Supplemental Table 1**. Twelve peripheral blood samples of healthy subjects with no history of influenza infection were used as controls.

### PBMCs Isolation and Cell Culture

PBMCs were isolated from whole blood using density centrifugation diluted 1:1 in a solution of human lymphocytes according to the manufacturer’s instructions (TBD, Tianjin, China). A549 and 16HBE cells were maintained in Dulbecco’s Modified Eagle’s Medium supplemented with 10% FBS, 100 U penicillin ml^-1^, and 100 mg streptomycin ml^-1^ at 37°C and 5% CO_2_. Jurkat T cell and PBMCs freshly isolated from peripheral blood were maintained in RPMI 1640 Medium supplemented with 10% FBS at 37°C and 5% CO_2_.

### Influenza Virus Culture, Propagation, and Infection

A/California/07/2009 (Influenza A H1N1 pdm09 virus) strain used in these studies was provided by the Chinese Center for Disease Control and Prevention (CDC, China). Virus was amplified for 2 days at 37°C in MDCK cells in DMEM (without FBS) supplemented with 2 μg/mL of tosylphenylalanyl chloromethyl ketone (TPCK)-trypsin (Sigma-Aldrich). Culture supernatant was aliquoted and immediately frozen at -80°C until used. Cells were seeded at appropriate density in 6-well plates and cultivated in growth medium until near confluence. Then cells were infected with viruses to obtain appropriate multiplicity of infection (MOI), cells were washed using PBS 1 ~ 2 hours after virus adsorption, and maintenance medium was added.

### ELISA, Chemical Reagents, and Inhibitors

The concentration of IL-36α, IL-36β, and IL-36γ in the plasma samples or cell supernatants was measured with Human IL-36 ELISA kit (R&D Systems) according to the manufacturer’s instructions. PGE2 levels in plasma were measured using the Prostaglandin E2 ELISA Kit (Elabscience), according to manufacturer’s instructions. IFN-α, IFN-β, and IFN-λ1 ELISA Kit was purchased from Neobioscience company (Shenzhen, China). Recombinant human IL-36α, IL-36γ, and IL-36Ra were purchased from R&D Systems. Polyinosinic-polycytidylic acid (Poly I:C) was purchased from Sigma-Aldrich. PGE2 and Etoricoxib were from Med Chem Express (MCE).

### Cell RNA Isolation, Reverse Transcription, and RT-PCR

Total cellular RNA was isolated using RNeasy Mini Kit (QIAGEN) according to the manufacturer’s instructions. The isolated RNA was then reverse transcribed into cDNA using RevertAid first strand cDNA synthesis kit (Thermo scientific) according to the manufacturer’s instructions. The Forgot-Me-Not™ Evagreen qPCR Master mix (Biotium) was used for quantitative PCR. Gene expression was normalized based on the expression of the glyceraldehyde-3-phosphate dehydrogenase gene (GAPDH). The primers used for PCR and transcription are listed in **Supplemental Table 2**. Relative fold changes in gene expression were determined using the threshold cycle (2–ΔΔCt) method. Relative levels of IAV-Ca07 Nucleoprotein (NP)-viral RNA (vRNA) and NP-mRNA were detected with reverse transcription primers and NP RT-PCR primers (**Supplemental Table 2**).

### Flow Cytometry Analysis

For analyzing IL-36R expression, PBMCs from patients and healthy individuals were purified using density centrifugation from whole blood. Indirect immunofluorescent staining was used by incubating PBMCs with anti-human IL-36R biotinylated antibody and normal goat IgG control biotinylated antibody (R & D Systems) at 4°C for 30 min in the dark. Then cells were washed and incubated with APC streptavidin antibody (TONBO biosciences), APC-H7 mouse anti-human CD3 Ab (BD Pharmingen™), BV605 mouse anti-human CD4 Ab, BV510 mouse anti-human CD8 Ab, BV650 mouse anti-human CD19 Ab (BD Horizon™), RedFluor™ 710 anti-human CD14 Ab, VioletFluor™ 450 anti-human CD11c Ab, FITC anti-human HLA-DR Ab (TONBO biosciences), or parallel isotype antibodies at 4°C for 30 min in the dark. After washing, the expression of cell surface molecules was analyzed using Flow Cytometry (Beckman Coulter, USA), and the results were expressed as the percentage or mean fluorescence intensity (MFI). A549,16HBE and Jurkat T cells were untreated or pre-treated with IL-36γ infected with IAV-Ca07 or left untreated for 24 h, and then cells were collected and subject to staining with anti-human IL-36R biotinylated antibody, normal goat IgG control biotinylated antibody at 4°C for 30 min in the dark. After washing, cells were incubated with APC streptavidin antibody. The expression of IL-36R in cell surface was analyzed by flow cytometry.

### Western Blotting and Image J Quantification

A549 and 16HBE cells were washed once with PBS, lysed in RIPA buffer (50 mM Tris, pH 7.6, 1% NP-40, 140 mM NaCl, 0.1% SDS), mixed with protease inhibitor Cocktail, PMSF solution (ROBY Biology), and Phos-STOP (Roche) in advance. Then the protein samples were resolved on 12% SDS-Polyacrylamide Gel Electrophoresis (PAGE) and transferred onto polyvinylidene fluoride membrane (GE Healthcare). The membrane was blocked with PBS containing 5% skim milk and probed with appropriate antibodies. Immunoblots were visualized using secondary antibodies conjugated to Chemiluminescent HRP substrate (ROBY Biology). Films were scanned and band volume intensity were measured using Image J software. The following reagents were used in western blotting: antibodies against human IL-36γ, LC-3B, p62, Mx1, PKR (Abcam), phosphorylated (p)-STAT1 (pY701), STAT1, (p)-STAT2 (pY141), STAT2, p-Akt, Akt, p-mTOR, mTOR, p-ULK1, ULK1, and GAPDH (Cell Signal Technology) and antibodies against IAV-Nucleocapsid (NP) and Matrix protein 1 (M1) (Sino Biological).

### Cell Apoptosis Analysis

A549 and 16HBE cells was pre-treated with IL-36γ or not and infected with IAV-Ca07 for 24 hours. Cells were then collected with 0.25% trypsin-EDTA, rinsed with pre-cooled PBS, and resuspended in binding buffer. Apoptosis was analyzed using the Annexin V-FITC apoptosis detection kit (BD Biosciences) according to the manufacturer’s instructions. Cells were then incubated for 15 min at room temperature in a dark condition. Subsequently, levels of apoptosis were evaluated using the flow cytometry and analyzed by Flow J software.

### Transmission Electron Microscopy (TEM)

A549 and 16HBE cells were seeded in 10-cm cell culture dishes and left untreated or pre-treated with 100 ng/ml IL-36γ for 12 h before cells infected with IAV-Ca07 for 12 h. The cells were digested and fixed in 4% glutaraldehyde and kept at 4°C for 24 h before washing 3 times with 0.1 M PBS. The cells were then post-fixed in 1% osmium tetroxide at 4°C for 2 h, followed by dehydrating in a graded series of ethanol and acetone. We then infiltrated, embedded, and polymerized the samples in ethoxyline resin. Ultrathin (60 nm) were prepared, stained with uranyl acetate and lead citrate, and then observed using a Hitachi Model JEM1230 transmission electron microscope. Photographs were taken using a Gatan-780 system.

### Statistical Analysis

All data are presented as the mean ± SD (standard deviation). Statistical analysis was performed using SPSS13.0 for Windows. Comparisons between the two groups were analyzed by paired Student’s t-tests. Comparisons among groups were made by one-way ANOVA test. Differences with P < 0.05 were considered statistically significant. All experiments were repeated at least three times.

## Results

### Expression of IL-36 Family Members and Other Cytokines in Plasma and PBMCs of Influenza-Induced ARDS Patients

**Figure 1:**
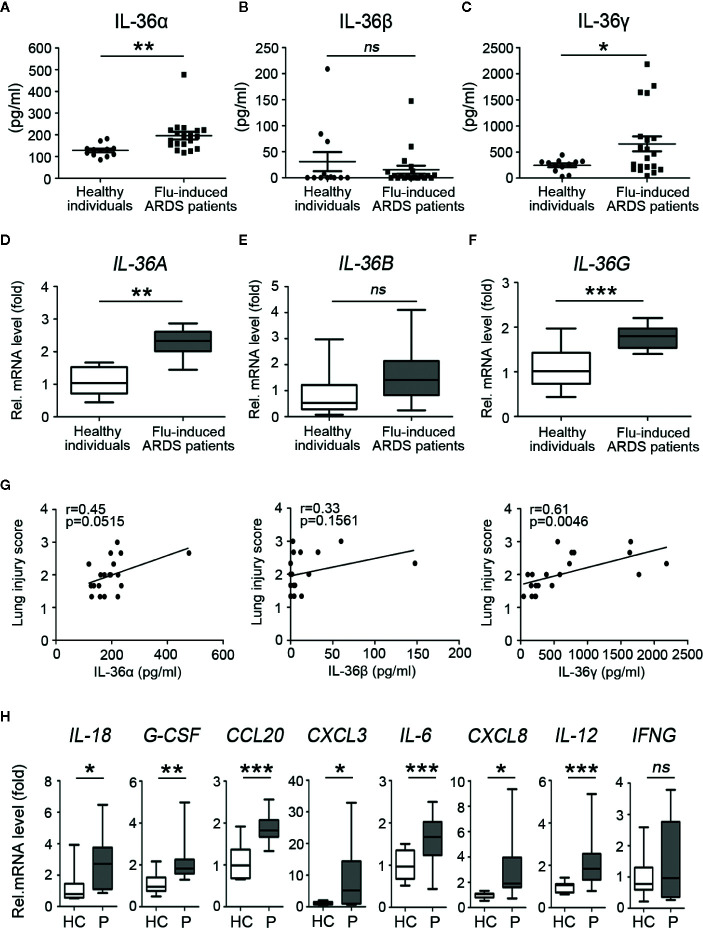
Upregulated expression of IL-36 family members and other cytokines in influenza-induced ARDS patients. Plasma and PBMCs were obtained from acute respiratory distress syndrome (ARDS) patients (*n* = 20) caused by influenza infection from day 0 to day 7 after onset and healthy individuals (*n* = 12). The concentration levels of **(A)** IL-36α, **(B)** IL-36β, and **(C)** IL-36γ in plasma (*n* = 20) were measured using ELISA. The relative mRNA expression levels of **(D)** *IL-36A*, **(E)** *IL-36B*, and **(F)** *IL-36G* in PBMCs (*n* = 18) were detected by RT-PCR. **(G)** The concentration levels of IL-36α, IL-36β, and IL-36γ in plasma of influenza-induced ARDS patients (*n* = 20) were analyzed, respectively, for the correlation with the lung injury score (LIS). **(H)** The relative mRNA expression levels of *IL-18*, *G-CSF*, *CCL20*, *CXCL3*, *IL-6*, *CXCL8*, *IL-12*, *IFNG* in PBMCs of flu-induced ARDS patients (*n* = 18) and healthy individuals (*n* = 12) were detected by RT-PCR. HC, Healthy controls; P, Patients. The data were shown as mean ± SD. Statistical significance is determined by Student’s t test. *P < 0.05, **P < 0.01, ***P < 0.001, ns, not significant.

To investigate and distinguish the role of IL-36 family members (IL-36α, IL-36β, IL-36γ) during influenza infection, plasma and peripheral blood mononuclear cells (PBMC) were isolated from healthy individuals and ARDS patients caused by influenza infection within 14 days from the onset of clinical symptoms (**Supplemental Table 1**). ELISA assay showed that levels of IL-36α, IL-36γ, but not IL-36β in plasma were significantly upregulated in patients, compared with healthy individuals (**Figure 1A–C**). A similar situation happened in the mRNA levels of *IL-36A* and *IL-36G* but not *IL-36B* PBMC from patients and healthy controls (**Figure 1D–F**). Simultaneously, the indices representing the severity of lung injury-lung injury score (LIS) were evaluated for the influenza-induced ARDS patients (18). LIS has a trend to be correlated with the IL-36α and IL-36β in the ARDS patients but with no statistical significance (IL-36α: *R* = 0.45, *P* = 0.0515; IL-36β: *R* = 0.33, *P* = 0.1561). However, IL-36γ was positively correlated with LIS (*R* = 0.61, *P* = 0.0046) (**Figure 1G**). Further RT-PCR analysis revealed that the mRNA levels of cytokines and chemokines that were regulated by IL-36 as previously described (19) were upregulated except for *IFNG* in PBMCs of IAV-infected patients (**Figure 1H**). Altogether, these data indicated that IL-36α/γ expression was upregulated after influenza infection, suggesting its potential function on immune regulation during influenza infection.

### Elevated Expression Profile of IL-36R in Human APCs, but Not T Cells of Influenza-Induced ARDS Patients

**Figure 2:**
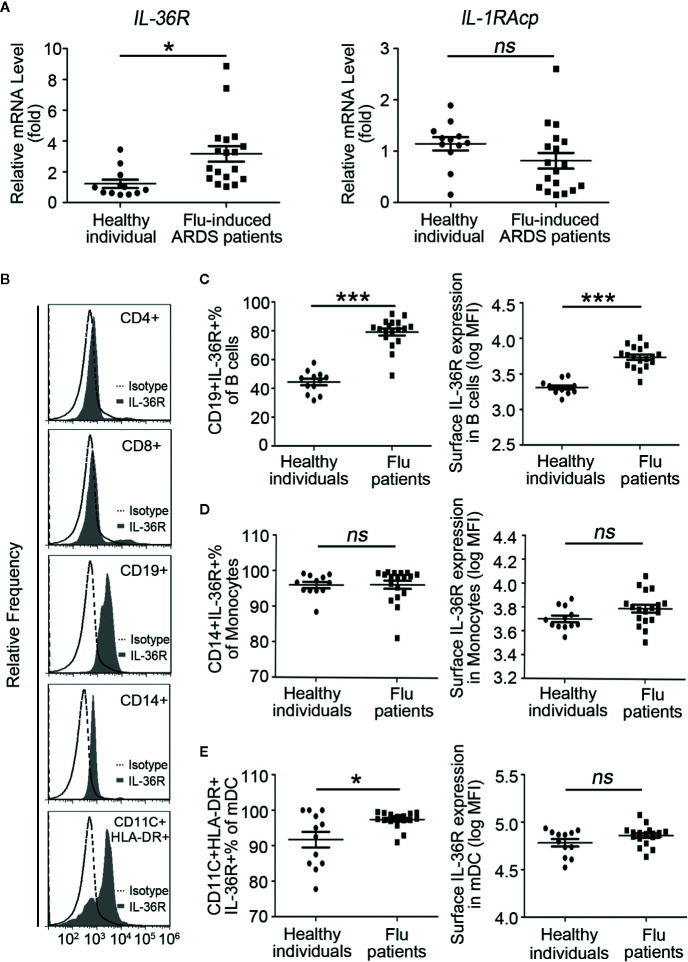
Elevated expression profile of IL-36 receptors in PBMCs of influenza-induced ARDS patients. **(A)** The relative mRNA expression levels of *IL-36R* and *IL-1RAcp* in PBMCs of influenza-induced ARDS patients (*n* = 18) and healthy individuals (*n* = 12) were detected by RT-PCR. **(B)** Flow cytometry to reveal IL-36R expression on the surface of T cells (CD4^+^ and CD8^+^), B cells (CD19^+^), monocytes (CD14^+^), and myeloid dendritic cells (mDCs, CD11c^+^HLA-DR^+^) in healthy individuals and histograms are representative of healthy donors. Filled histogram: anti-IL-36R; Dotted histogram: isotype control Ab. The proportion of **(C)** IL-36R^+^ B cells in total B cells, **(D)** IL-36R^+^ monocytes in total monocytes, and **(E)** IL-36R^+^ mDCs in total mDCs from patients (*n* = 18) and healthy individuals (*n* = 12) were detected using flow cytometry. **(C–E)** The expression level of IL-36R on B cells, monocytes, and mDCs from patients (*n* = 18) and healthy individuals (*n* = 12) were detected using flow cytometry. Results are presented as scatter plots with median of the proportion or mean fluorescence intensity (MFI) subtracting corresponding isotypic controls. Statistical significance is determined by Student’s t test. *P < 0.05, ***P < 0.001, ns, not significant.

Because IL-36 family cytokines function through binding to IL-36R and the recruitment of the co-receptor IL-1RAcP, we have to test expression of IL-36R and IL-1RAcp in both influenza related ARDS patients and normal individuals. RT-PCR results showed that the mRNA levels of *IL-36R*, but not *IL-1RAcP*, were significantly higher in all flu patients than healthy individuals (**Figure 2A**; **Supplemental Figure 1A**). Considering the prominent enhancement of IL-36 and its receptor in flu patients, we explored what kind of cells would respond to IL-36 cytokines. T cells, B cells, monocytes, and myeloid dendritic cells (mDCs) from healthy individuals were stained with Abs against IL-36R and analyzed using flow cytometry. Detailed process of flow cytometry analysis was shown in **Supplemental Figures 1B, C**. Results showed that IL-36R was absent from the surface of T cells, including CD4^+^ T cells and CD8^+^ T cells; however, it was most strongly expressed on the surface of mDCs and partially expressed on the surface of monocytes and B cells from a healthy individual (**Figure 2B**). Next, to explore whether IL-36R expression varies between flu patients and healthy individuals, we measured IL-36R expression in immune cells and results showed significantly higher IL-36R^+^ B cells % in pool of total B cells and elevated surface IL-36R expression in total B cells (**Figure 2C**; **Supplemental Figure 1E**). The representative dot plots of IL-36R^+^ B lymphocytes are shown in **Supplemental Figure 1D**. However, by comparing with healthy individuals, we didn’t find statistically significant difference in IL-36R^+^ monocytes % in pool of total monocytes and surface IL-36R expression in total monocytes in influenza patients (**Figure 2D**). Similarly, no major difference on surface IL-36R expression in mDCs but slightly upregulated IL-36R^+^ mDCs % in pool of total mDCs were presented in flu-induced ARDS patients (**Figure 2E**). Taken altogether, these data demonstrated the elevated expression profile of IL-36R in various immune cells among influenza-induced ARDS patients.

### Upregulation of IL-36γ in Respiratory Epithelial Cells and PBMCs Needs Stimulation of Live IAV or Poly I:C

**Figure 3:**
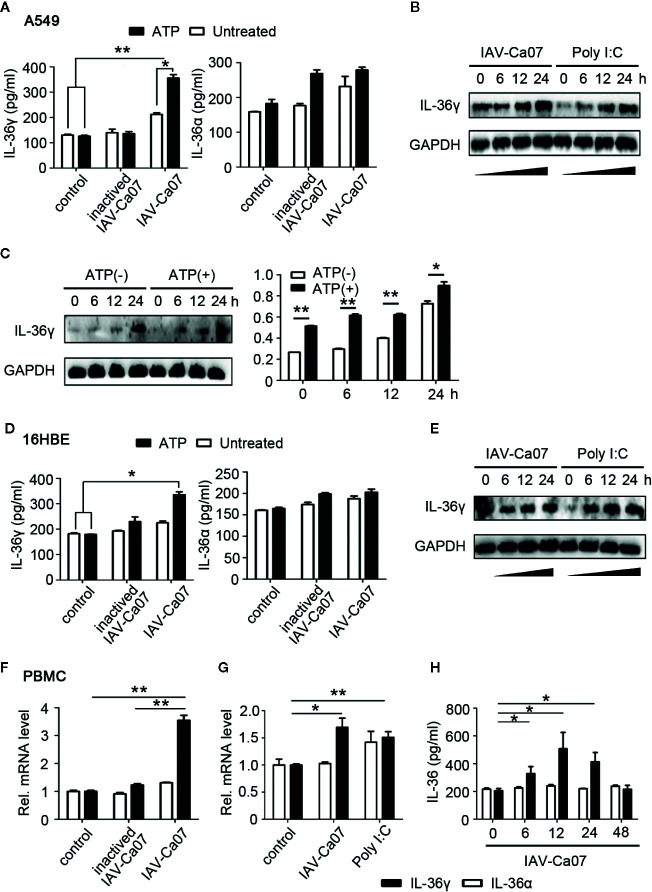
IAV induced IL-36 expression in different cell types. **(A)** A549 cells and **(D)** 16HBE cells were infected with ultraviolet (UV)-inactivated IAV or IAV (H1N1, A/California/07/2009) (MOI = 1). After 12 h incubation, A549 were treated with or without ATP (1 mM) during 2 h of incubation, and then the supernatants of cell culture were harvested. The protein levels of IL-36γ and IL-36α were detected using ELISA. **(B)** A549 cells and **(E)** 16HBE cells were infected with IAV (MOI = 1) or stimulated with poly I:C (100 μg/ml), an analogue of the viral replication intermediate double-stranded RNA. Levels of IL-36γ were determined by western blot for the indicated timeframes. **(C)** A549 cells were infected for the indicated timeframes with IAV (MOI = 1), and after this treated with or without ATP (1 mM, 2 h). Intracellular protein level of IL-36γ was examined and analyzed by western blotting and Image J. **(F)** PBMCs were infected with UV-inactivated IAV or IAV for 12 h. *IL-36A* and *IL-36G* mRNA levels were quantified using RT-PCR. **(G)** PBMCs were infected with IAV or stimulated with poly I:C (100 μg/ml) for 12 h. *IL-36A* and *IL-36G* mRNA levels were quantified using RT-PCR. **(H)** PBMCs were infected for the indicated timeframes with IAV (MOI = 1) and treated with ATP (1 mM, 2 h), the protein levels of IL-36α and IL-36γ were quantified using ELISA. Data were shown as mean ± SD of three independent experiments. Statistical significance is determined by Student’s t test. *P < 0.05, **P < 0.01.

Next, we questioned whether IL-36 expression in human respiratory epithelial cells and PBMCs needs the stimulation of live virus. A549 cells were infected with IAV-Ca07 or incubated with heat-inactivated IAV, and IL-36 in culture supernatants was qualified using ELISA. Results showed that IL-36γ expression was upregulated in epithelial cells infected with live influenza virus, compared with non-infected controls. However, inactivated virus stimulation didn’t significantly increase IL-36α protein secretion (**Figure 3A**). Previous studies indicated that extracellular adenosine triphosphate (ATP) was a significant mediator for extracellular secretion of IL-36 cytokines (12). Thus, ATP (1 mM) was added after 12 h of infection and ELISA results showed IL-36γ increased to some extent (**Figure 3A**). Western blotting results revealed that intracellular protein levels of IL-36γ increased as well when ATP was existing, and in a time-dependent manner (**Figure 3C**). In addition, A549 cells were stimulated with toll-like receptor 3 (TLR3) ligand poly I:C, a double-stranded RNA mimic that can be used as an immune stimulant. And western blotting results showed that both IAV-Ca07 infection and poly I:C treatment up-regulated IL-36γ expression (**Figure 3B**). To test whether this phenomenon occurred in 16HBE cells, we conducted similar experiments. ELISA results showed that IAV-Ca07 infection induced IL-36γ but not IL-36α protein expression, and additional ATP treatment increased IL-36γ expression, albeit to a lesser extent (**Figure 3D**). Consistently, western blotting results indicated that both IAV-Ca07 infection and poly I:C stimulation also increased IL-36γ expression compared with controls (**Figure 3E** and **Supplemental Figure 2A**). Similar experimental methods were applied to PBMC from a healthy donor. RT-PCR results showed that IAV-Ca07 infection and poly I:C stimulation of freshly isolated PBMCs significantly increased IL-36γ expression compared with no-infection control (**Figure 3F, G**). The PBMCs infected with IAV-Ca07 expressed and secreted IL-36γ but not IL-36α in a time-dependent manner. IL-36γ was significantly upregulated at 6 h post-infection and peaked at 12 h (**Figure 3H**). Altogether, these data indicated that IL-36γ can be induced effectively in both human respiratory epithelial cells and immune cells after IAV infection and poly I:C stimulation.

### COX-2/PGE2 Pathway Is Involved in IAV-Ca07-Induced IL-36γ/α mRNA Expression

**Figure 4:**
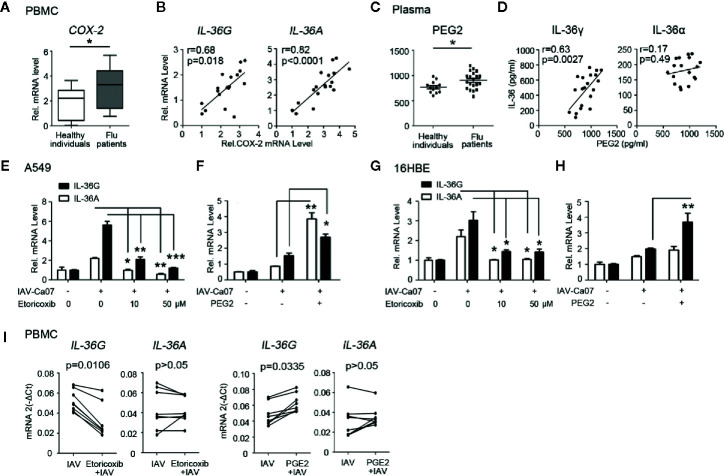
COX-2/PGE2 pathway is involved in IAV-induced IL-36α/γ expression. **(A)** The relative mRNA expression levels of *IL-36R* and *IL-1RAcp* in PBMCs of influenza-induced ARDS patients (*n* = 18) and healthy individuals (*n* = 12). **(B)** Relative *COX-2* and *IL-36G* or *IL-36A* mRNA levels in PBMCs from influenza patients were subjected to Pearson correlation analysis (*n* = 18). **(C)** The concentration levels of PGE2 in plasma of influenza-induced ARDS patients (*n* = 18) and healthy individuals (*n* = 12) were measured using ELISA. **(D)** Concurrent detection of PGE2 and IL-36γ or IL-36α in serum samples of influenza-induced ARDS patients (*n* = 18). Graph shows a correlative relationship between serum IL-36 and PGE2. **(E)** A549 cells and **(G)** 16HBE cells were incubated with the indicated concentrations of Etoricoxib for 3 h, and infected with IAV (MOI = 1) for 12 h, then subjected to RT-PCR analysis of *IL-36G* and *IL-36A* expression. **(F, H)** Experiments performed as in **(E, F)**, except 10 μM PGE2 was added. Data were shown as mean ± SD of three independent experiments. **(I)** PBMC from healthy individuals (*n* = 8) were incubated with Etoricoxib (50 μM) or PGE2 (10 μM) for 3 h, and infected with IAV (MOI = 1) for 12 h, then subjected to RT-PCR analysis of *IL-36G* and *IL-36A* expression. Statistical significance is determined by Student’s t test in all experimental results. *P < 0.05, **P < 0.01, ***P < 0.001.

COX-2/PGE2 signaling axis can partly mediate inflammatory response induced by viral infection (20). To verify COX-2 correlation with IL-36 generation caused by influenza infection, PBMCs were isolated from whole blood of influenza patients and healthy donors and then subjected to RT-PCR analysis to investigate expression of COX-2 and PGE2 levels in clinical samples. Results showed that mRNA level of COX-2 was significantly upregulated in influenza patients compared with healthy individuals (**Figure 4A**). Further analysis revealed that elevated level of *IL-36G* and *IL-36A* were statistically correlated with *COX-2* in the influenza patients (*IL-36G*: *R* = 0.68, *P* = 0.0018; *IL-36A*: *R* = 0.82, *P* < 0.0001; **Figure 4B**). To verify the relationship between IL-36 and PGE2 during IAV infection, we compared the levels of PGE2 in serum of two groups, and we found that the influenza patients were slightly elevated (**Figure 4C**). The levels of IL-36γ, IL-36α, and PGE2 were measured and analyzed using Pearson’s correlation. Result showed that serum IL-36γ but not IL-36α levels were positively correlated with serum PGE2 levels (*IL-36G*: *R* = 0.63, *P* = 0.0027; **Figure 4D**). These data acquired from clinical samples suggested that IL-36γ/α expression was significantly associated with the COX-2/PGE2 pathway.

Since there is significant correlation between the IL-36γ/α and COX-2/PGE2 pathway, we thus detected the roles of COX-2/PGE2 pathway in IAV induced IL-36γ/α mRNA expression in respiratory epithelial cells. A549 cells were pre-treated with Etoricoxib, a selective COX-2 inhibitor, for 3 h at different doses before infected with IAV-Ca07. We observed significantly decreased IL-36γ and IL-36α mRNA levels in a dose-dependent manner after pretreatment with Etoricoxib (**Figure 4E**). Moreover, IL-36γ/α mRNA expression increased after addition of PGE2 (**Figure 4F**). Similar experiments were conducted in 16HBE cells. Results showed that IL-36γ/α mRNA levels decreased in a dose-dependent manner with pretreatment with Etoricoxib (**Figure 4G**). However, PGE2 only increased IL-36γ mRNA expression but not IL-36α (**Figure 4H**).

We next evaluated whether COX-2/PGE2 pathway acted as a mediator on the expression of IL-36 in human PBMCs. PBMCs were isolated from eight healthy individuals and incubated with COX-2 inhibitor Etoricoxib and PGE2 for 3 h. After IAV-Ca07 infection for 12 h, we found that the mRNA levels of IL-36γ but not IL-36α decreased after pretreatment with Etoricoxib, and increased when incubated with PGE2 (**Figure 4I**).

### Influenza A Virus Stimulation Can Effectively Increase IL-36R Expression

IL-36 members functioning as inflammatory regulator depend on the presence of IL-36 receptors on the cell surface (13). To substantiate the expression of IL-36R in various model cells, we utilized flow cytometry to quantify IL-36R expression on the surface of Jurkat T cells. Results showed that IL-36R was absent from the surface of Jurkat T cell, no matter if there was IL-36γ and IAV-Ca07 stimulation or not (**Supplemental Figure 3A**). Then we used Jurkat T cell lines as a negative control to evaluate IL-36R mRNA expression in A549 and 16HBE cell lines. RT-PCR results showed that IL-36R expression was readily detected in A549 and 16HBE cells relative to Jurkat T cells. However, IL-1Acp expression was significantly higher in Jurkat T cells than others (**Supplemental Figure 3B**). Considering that cellular typing and biochemical analysis of bronchoalveolar lavage fluid (BALF) can provide critical information regarding the disease state of influenza patients, we plan to figure out expression differences of IL-36R and IL-1RAcp between clinical BALF and blood samples. BALF and corresponding PBMCs samples from three influenza patients were collected and total mRNA was extracted and then subjected to analysis of IL-36R and IL-1RAcp expression. RT-PCR results showed that level of IL-36R, but not IL-1RAcp mRNA, was significantly higher in BALF than PBMCs (**Supplemental Figure 3C**).

**Figure 5:**
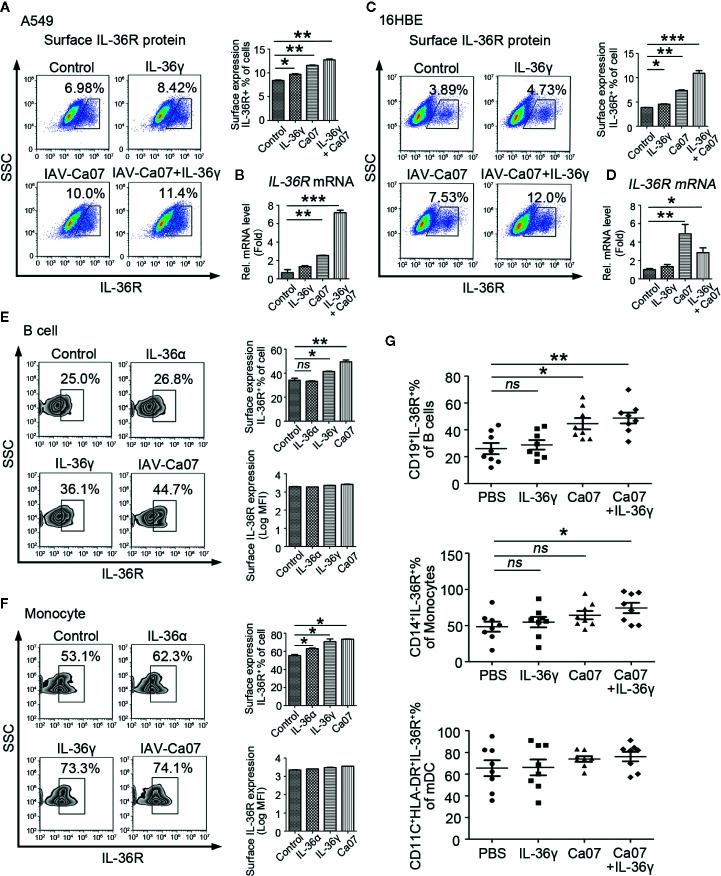
Influenza virus can effectively increase IL-36R expression. IL-36R expression on the surface of A549 and 16HBE cells was determined by flow cytometry **(A, C)** and RT-PCR **(B, D)** after stimulated with IL-36γ (100 ng/ml) or infected with IAV (MOI = 1) for 24 h. **(E, F)** Representative flow cytometric analysis of IL-36R expression on B cells and monocytes after infected with IAV (MOI = 1) or stimulated with IL-36γ and IL-36α (each 100 ng/ml) for 24 h. **(G)** The proportion of IL-36R^+^ B cells, monocytes, and mDCs in respective total cell pools from healthy donors (*n* = 8) after infected with IAV (MOI = 1) or stimulated with IL-36γ (100 ng/ml) for 24 h were analyzed statistically according to flow cytometry results. Results are presented as scatter plots with median of the proportion subtracting corresponding isotypic controls. Statistical significance is determined by Student’s t test. *P < 0.05, **P < 0.01, ***P < 0.001, ns, not significant.

To investigate whether IL-36γ or IAV-Ca07 stimulation was involved in upregulated surface IL-36R expression, A549 and 16HBE cells were stimulated with IL-36γ and IAV-Ca07 individually or simultaneously. Upon being stimulated for 24 h, cells were stained with Abs against IL-36R and analyzed by flow cytometry, and results showed that surface IL-36R^+^ A549 and 16HBE cell % in total cells had a moderate increase following IL-36γ and IAV-Ca07 treatment (**Figures 5A, C**). In addition, IAV-Ca07 and its combination with IL-36γ mediated upregulation of IL-36R mRNA was readily observed in both cell types (**Figure 5B, D**).

Similar results were obtained in PBMCs after IAV-Ca07 infection. B cells and monocytes from healthy individuals were stimulated with IL-36α, IL-36γ, and IAV-Ca07 and then stained with Abs against IL-36R. Flow cytometry results showed that the percentage of surface IL-36R^+^ cells in total B cells and monocytes significantly increased in the presence of IL-36γ and IAV-Ca07. However, no significant difference was observed on the surface IL-36R expression quantity in total B cells and monocytes (**Figure 5E, F**). To substantiate the laboratory result, PBMCs from eight healthy donors stimulated with IL-36γ or IAV-Ca07 individually and simultaneously. The percentage of IL-36R^+^ cells in B cells increased in the presence of IAV-Ca07 or combined with IL-36γ; IL-36R^+^ cells in pool of monocytes had an obvious increase under the stimulation of IAV-Ca07 combined with IL-36γ. However, there was no obvious change of IL-36R^+^ cells in mDC, and even joint stimulus existed (**Figure 5G**). These data demonstrated that IAV stimulation could effectively increase IL-36R expression.

### IL-36γ Enhance IFNs Production but Promote IAV-Mediated IFN-Stimulated STAT1 and STAT2 Phosphorylated Inhibition in Lung Epithelial Cells

**Figure 6:**
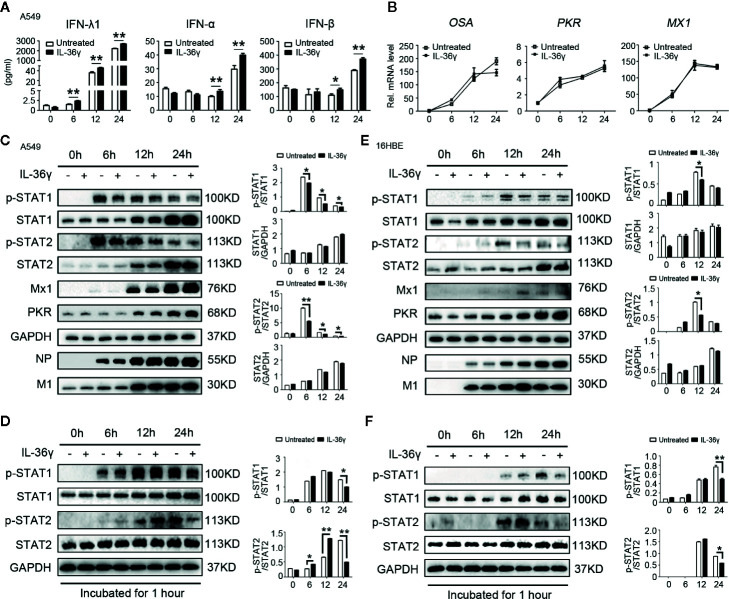
IL-36γ enhance IFNs production and promote IAV-mediated IFN-stimulated STAT1 phosphorylated inhibition in lung epithelial cells. **(A)** A549 cells were treated with IL-36γ (100 ng/ml) or left untreated 24 h prior to infection with IAV-C07 (MOI = 1). Levels of IFN-α, IFN-β, and IFN-λ1 in supernatant were measured by ELISA in different timeframes, as indicated. **(B)** Experiments performed as in **(A)**, except levels of IFN-stimulated genes (ISGs), such as OSA, PKR, and Mx1, were quantified using RT-PCR. **(C)** Simple medium only and IL-36γ pre-treated A549 cells were further infected with IAV-Ca07 (MOI = 1), then cells were collected and lysed at the indicated time, and the levels of p-STAT1 and p-STAT2, non-phosphorylated STAT1 and STAT2, Mx1, PKR, IAV-Ca07 NP, and M1 proteins were assessed by western blotting and Image J. **(D)** A549 cells were stimulated by supernatant culture medium from IAV-infected cells in **(C)**, followed by western blotting with indicated antibodies. **(E, F)** Experiments performed as in **(C, D)**, except 16HBE cells used. The data were shown as mean ± SD. Statistical significance is determined by Student’s t test. *P < 0.05, **P < 0.01.

Considering that potential role of IL-36γ, as a proinflammatory cytokine plays in influenza virus-related immune response, we examined whether IL-36γ could enhance antiviral activity associated with production of IFNs and ISGs after influenza infection. A549 cells were pre-treated with IL-36γ for 12 h or not before IAV-Ca07 infection and subjected to IFNs level quantification. ELISA results showed that levels of IFN-λ1, IFN-α, and IFN-β dramatically increased after IAV-Ca07 infection in a time-dependent manner, and cells pre-treated with IL-36γ resulted in even higher levels of IFNs (**Figure 6A**). To make clear the modes of interaction *in vitro*, A549 cells were pre-treated with IL-36R receptor antagonist (IL-36Ra) prior to IAV challenge. The results showed that IFNs decreased significantly when cells pre-treated with IL-36γ combined with IL-36Ra before IAV-Ca07 infection (**Supplemental Figure 4B**). However, no significant increase was observed in the levels of *PKR*, *OSA*, and *MX1* mRNA in cells pre-treated with IL-36γ (**Figure 6B**). Consistently, pre-treatment with IL-36γ in A549 cells didn’t strongly decrease IAV replication (**Supplemental Figure 4A**). Wei et al. showed that influenza virus can suppress interferon-stimulated STAT phosphorylation and affect antiviral response in the host (21). To further explain the finding above, downstream factors of interferon signaling pathway were detected using western blotting. We found that phosphorylation of STAT1 and STAT2 in A549 cells pre-treated with IL-36γ was inhibited at each post-infection point in time, while no significant changes in Mx1, PKR, IAV-Ca07 NP, and M1 protein were observed (**Figure 6C**). To substantiate the phenomenon, corresponding culture supernatants from the infected A549 cells left untreated or pre-treated with IL-36γ were collected. Another prepared A549 cells were subsequently incubated with these culture supernatants for 1 h. We found that level of STAT1 and STAT2 phosphorylation in IL-36γ pre-treated cells was dramatically decreased at later stages (at 24 h) of infection (**Figure 6D**). These data suggested that the IL-36γ negatively affected IFN-stimulated STAT1 and STAT2 phosphorylated in A549 cells after IAV infection.

Next, we questioned whether these phenotypes exist in IAV-stimulated bronchial epithelial cells. Experiments were performed as above in 16HBE cells. Results showed that only lower IFN-λ1 level in the supernatants increased in IAV-Ca07 infected cells, and cells pre-treated with IL-36γ resulted in relatively higher levels of IFN-λ1 12 h and 24 h after IAV-Ca07 infection (**Supplemental Figure 2B**). Likewise, phosphorylation of STAT1 and STAT2 in 16HBE cells pre-treated with IL-36γ was mildly inhibited at 12 h after IAV-Ca07 infection, while no significant changes in Mx1, PKR, IAV-Ca07 NP, and M1 protein were observed (**Figure 6E**). In addition, we also found that the level of STAT1 and STAT2 phosphorylation was decreased when incubated with culture supernatants of 16HBE cells pre-treated and IL-36γ pre-treated (**Figure 6F**).

Overall, these results confirmed that IL-36γ could affect interferon signaling pathway through not only augmenting interferon production but also promoting IAV-mediated IFN-stimulated STAT1 and STAT2 phosphorylated inhibition, which could attenuate innate anti-virus immune response.

### IL-36γ Promotes Apoptosis and Inhibits Autophagy in Lung Epithelial Cells With IAV-Infection

**Figure 7:**
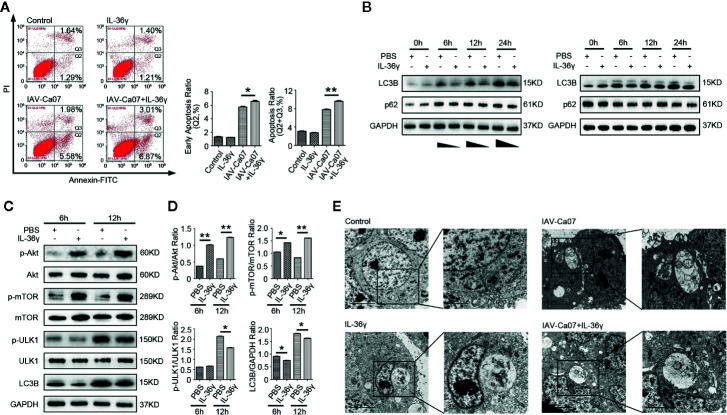
IL-36γ promote apoptosis and inhibit autophagy in IAV-infected lung epithelial cells. **(A)** A549 cells were infected with or without IAV-Ca07 (MOI = 1) and treatment with or without IL-36γ, and apoptotic cells were determined by flow cytometry. The early apoptosis ratio and total apoptosis ratio was analyzed using Flow J software. **(B)** Western blotting analysis of endogenous LC-3B and p62 protein in A549 cells upon infected with or without IAV-Ca07 and treatment with or without IL-36γ at indicated time points. **(C)** Western blotting analysis of Akt, mTOR, ULK1, and LC-3B in A549 cells infected with IAV-Ca07 and treatment with IL-36γ (100 ng/ml) for 6 h and 12 h. **(D)** The relative expression of Akt, mTOR, ULK1, and LC-3B was consistent with that shown in **(C)**. **(E)** Transmission electron microscopy (TEM) analysis showing the formation of autophagosomes in A549 cells after infected with or without IAV-Ca07 and treatment with or without IL-36γ for 12 h. Representative autophagosomes or autolysosomes (black arrows) in A549 cell are highlighted in the enlarged images (right). The data were shown as mean ± SD. Statistical significance is determined by Student’s t test. *P < 0.05, **P < 0.01.

To investigate whether IL-36γ affected cell apoptosis after IAV infection, A549 cells were infected with or without IAV-Ca07 and then treated with or without IL-36γ. Flow cytometry results showed that IL-36γ promoted apoptosis in IAV-Ca07 infected A549 cells, with higher levels being presented in the late stage (**Figure 7A**). However, IL-36γ promoting apoptosis in IAV-infected 16HBE cells only presents in late stages (**Supplemental Figure 2C**). Wang et al. reported that influenza virus can induce autophagosome accumulation and autophagy promotes replication of Influenza virus in turn (22). Pharmacological inhibition of autophagy could decrease influenza viral yields (23). To clarify the relationship between IL-36γ and autophagy in infected A549 cells, we investigate the expression of LC-3B protein, an accurate indicator for determining autophagy, after viral infection and IL-36γ treatment in indicated time points. Western blotting results showed that IL-36γ-treated A549 cells had a decreased LC-3B protein level compared with untreated cells after influenza infection. However, levels of LC-3B protein decreased mildly compared with blank control (**Figure 7B**). The expression of phosphorylated (p)-Akt, p-mTOR were significantly increased and p-ULK1 were decreased in IAV-Ca07 infected A549 cells treated with IL-36γ. Similarly, the LC-3B-to-GAPDH ratio relative to controls was decreased (**Figure 7C, D**). We also found widely distributed small mobile autophagosomes, and large vesicles appeared near the perinuclear cavity in these cells using transmission electron microscopy (TEM). However, we observed fewer vesicles inside the cells and less membrane vacuoles in IL-36γ-treatment A549 cells after IAV infection. Few autophagosomes can be observed in the cytoplasm of non-infected A549 cells (**Figure 7E**). In addition, TEM showed decreased intracellular autophagosomes in IL-36γ-treatment 16HBE cells after IAV infection (**Supplemental Figure 2D**). Overall, these results demonstrated that IL-36γ could promote apoptosis and inhibit autophagy in IAV-infected lung epithelial cells.

## Discussion

In our study, we revealed the detailed expression pattern of IL-36 and its subtypes, as well as the distribution and expression level of IL-36R in immune cells in severe influenza patients, which indicated that IL-36γ expression might change or exert specific immunological functions during influenza infection. In addition, we also demonstrated a possible route that produces IL-36γ, potential induction mechanism, and variation of IL-36R after IAV infection. Furthermore, we observed that IL-36γ could effectively regulate interferon-related signaling pathway and control the progress of PCD *via* promoting apoptosis and inhibiting autophagy in the early stages of infection, suggesting its auxiliary role in innate immunity to influenza infection.

It has been reported that IL-36γ or IL-36α significantly upregulated in patients with active pulmonary tuberculosis and bacterial pneumonia (11, 24). In our study, we observed higher concentrations of IL-36γ in plasma and elevated mRNA levels of *IL-36G* and *IL-36R* in influenza-induced ARDS patients, suggesting its potential association with viral infection. Previous research had shown that mouse naive CD4^+^ T cell can express IL-36R and respond to IL-36 effectively, and IL-36 together with IL-12 can promote murine Th1 polarization (25). However, unlike in mouse, IL-36R protein partially expressed in human APC, including monocytes and mDCs, but not T cells, which is consistent with previous study (26, 27). The relatively low rate of viral RNA clearance and prolonged viral shedding observed in severe influenza may lead to long-term viral burden and hyper-inflammation induction in patients, which can be also considered as “persistent infections and chronic stimuli” status (28). Therefore, it is reasonable to infer that the higher expression of IL-36R in the APC during influenza infection is essential stress feedback of the host and contributes to adapting to the “long-term persistent infection.” It is worthwhile to note that IL-36R protein can also be detected in the surface of B lymphocytes, and the percentage of IL-36R^+^ B cells in B cells pool in severe influenza patients is about 40% higher than controls. B lymphocytes are generally thought to mediate the humoral immunity response by generating and secreting antibodies; however, the immunomodulatory effects, especially in viral infection, have not been fully recognized (29). Our findings suggested that, during viral infection, initiation of IL-36/IL-36R signaling axis in B cell may also be involved in modulating the balance of virus-induced inflammatory response. Therefore, the elevated levels of IL-36/IL-36R may suggest its potential role in pro-inflammatory pathogenesis of severe influenza infection.

Compared with other cytokines, the secretion of IL-36 family members follows a non-classical secretion route, the endoplasmic reticulum/Golgi independent unconventional protein secretion pathway (30). Previous studies showed that intracellular IL-36γ release requires coordinated stimulus of extracellular ATP, which is beneficial to the activation of NLRP3 inflammasome (13, 30). Indeed, our present results also demonstrated that ATP treatment could increase and control IL-36γ expression and secretion in human respiratory epithelial cells, indicating their probable contribution in the influenza-induced activation of inflammasome and inflammatory signaling pathways. It has been reported that IL-36 cytokines are synthesized as precursor proteins with little bioactivity and then activated *via* cleavage at different proteolytic sites (31). We found the level of IL-36γ induced by IAV-Ca07 in PBMCs was higher than that in epithelial cells. One possible explanation is that IL-36γ might be directly released from immune cells and subsequently cleaved by neutrophil-derived proteases in blood for maximal activation instead of precursor forms released from epithelial cells. Consistent with previous reports, we also found that IL-36γ rather than IL-36α in mice lung tissue appears inducible after IAV-Ca07 infection, despite only 2~3-fold induction at transcript level (data not shown). Overall, these results implied the significant role of IL-36γ in influenza infection.

Given COX-2/PGE2 signaling axis was involved in inflammatory response induced by viral infection, we hypothesized that it exerts influence in IL-36 induction (32). Indeed, we found that expressions of COX-2 and PGE2 were positively correlated with IL-36 in blood samples from severe influenza patients. Research of high-throughput screen from influenza patients reported that many proteins involved in the immune response were regulated by COX-2 (33). However, no data of IL-36 in the inflammatory cascade mediated by the pro-inflammatory factor COX-2 was detected. Our results indicated that COX-2/PGE2 signaling axis may be involved in IAV-induced IL-36 expression in lung epithelial cells as a forward regulator or partner factor. Clear location relationship between COX-2/PGE2 signaling axis and IL-36 release would contribute to identify appropriate targets of anti-inflammatory drugs with less adverse effects. Therefore, the cross-talk, including feedback regulation mechanisms, between COX-2/PGE2 pathway and IL-36 expression *in vivo* and *in vitro* requires further investigation.

Previous reports elucidated that the expression of IL-36R was slight in monocytes but increased when they differentiated into macrophages (34). Our results also confirmed this conclusion through comparing peripheral blood with BALF (major components: alveolar macrophages) and showed that mRNA level of *IL-36R* in BALF was higher than in PBMCs. Moreover, it has been reported that IL-36α could induce neutrophil influx and increase the expression of IL-36R in the lung tissues of mice (35). Our current study demonstrated that IL-36γ and influenza viruses were also an important inducer to promote expression of IL-36R in respiratory epithelial cells and immune cells isolated from whole blood.

IL-1 family were considered as vital adjuvants for the activation of mucosal immunity against IAV infection, indicating that they could act as co-enhancers or co-mediators of the immune response (36). Indeed, mucosal innate immune system brought a stronger barrier to IAV infections; however, release of large amounts of proinflammatory cytokines accelerates the disease process of IAV-triggered damage to lung tissues (5, 37). IFN-λ but not IFN-α/β was considered as the primary IFNs product that acts at the airway epithelial barrier to limit initial viral infection without activating obvious inflammation (38). Our findings also demonstrated significant increase of IFN-λ1 compared with type I IFNs in infected alveolar epithelial cells. And additional IL-36γ could enhance IFNs production. Normally, as an essential antiviral cytokine, IFNs can trigger production of ISGs, which directly inhibits viral replication and degrades viral nucleic acids (39). However, viruses, including IAV, have developed several mechanism to escape the host immune response, such as inhibiting IFNs production and interfering IFNs signaling pathway (40, 41). Our findings show that although the induction of IFNs increased in present of extra IL-36γ, the corresponding ISGs expression was not affected. And it is further confirmed that alveolar epithelial cells pre-treated with IL-36γ didn’t strongly inhibit IAV replication after infection (data not shown). Previous reports showed that IAV negatively regulated JAK-STAT signaling pathway *via* inhibition of IFN-λ stimulated STAT1 phosphorylation or induction of NF-κB dependent SOCS-3 expression in epithelial cells (21, 42). Here, we identified negative regulation effect of IL-36γ *via* promoting IAV-mediated IFN-stimulated STAT1 and STAT2 phosphorylated inhibition in lung and bronchial epithelial cells. This suggested that although raised IFNs induced by IL-36γ presented as a defense mechanism, viral immune evasion also enhanced concomitantly, which can be regarded as “bidirectional influence.” Overall, IL-36γ might play multiple regulatory roles in classic interferon signaling cascades.

As an essential regulatory mechanism, apoptosis and autophagy play an important role in host protection within airway epithelium during influenza infection (43–45). Apoptosis exerts pleiotropic functions, such as attenuating lung immunopathologic damage by hindering invading and virus replication. However, autophagy promotes influenza virus replication. Thus, it was early autophagy blocking or later autophagosome degradation that might bring one avenue to impair influenza virus infection and replication (43, 46, 47). Our findings showed that IL-36γ could positively regulate antiviral immunity within epithelial cells *via* controlling the progress of programmed cell death, including promoting apoptosis and limiting autophagy. Previous reports showed that LC-3B, especially LC-3BII, accumulated in a few hours of post-infection in A549 cells (48). Our results indicated that IL-36γ can delay early accumulation of LC-3B *via* activating Akt/mTOR/ULK1 pathway, but exert few effects on synthesis of sequestosome (p62), a multifunctional scaffold protein involved in autophagosome degradation. This suggested that IL-36γ exerts antagonism functions to some extent for influenza virus-induced incomplete autophagy instead of integrated autophagy process. Overall, although it didn’t control virus replication in lung epithelial cell, IL-36γ might increase cellular sensitivity to anti-virus immunity by influencing process of programmed death.

In conclusion, we find that IL-36γ is a critical host immune factor in inflammatory conditions of the lung and have significant modulatory functions in balancing antiviral response and viral immune evasion of human airway epithelial cell. Our study thereby provides a new application foreground for evaluation of clinical immune status using IL-36γ during influenza infection. It also brings new clues for elucidation of immunomodulatory mechanism of IL-36γ-mediated anti-viral responses in pulmonary disorders.

## Data Availability Statement

The original contributions presented in the study are included in the article/**Supplementary Material**; further inquiries can be directed to the corresponding author.

## Ethics Statement

The studies involving human participants were reviewed and approved by China-Japan Friendship Hospital Ethics Committee (Approval No. 2018-120-K86). The patients/participants provided their written informed consent to participate in this study. Written informed consent was obtained from the individual(s) for the publication of any potentially identifiable images or data included in this article.

## Author Contributions

SL and BC conceived the project. SL, HL, YMW, HBL, SSD, XHZ, XLZ, and BC discussed and designed the experiments. SL, HL, and SD conducted the experiments. LS and HL performed sequencing and statistical analyses. LS and BC wrote the manuscript. All authors contributed to the article and approved the submitted version.

## Funding

This work funded by the Natural Science Foundation of China (NO. 81970010/H0104), National Key Research and Development Program of China (2018YFC1200102), the Fundamental Research Funds for the Central Universities and Research projects on biomedical transformation of China-Japan Friendship Hospital (PYBZ1820), the Ministry of Science and Technology of China (2017ZX10103004), and the CAMS Innovation Fund for Medical Sciences (CIFMS 2018-I2M-1-003).

## Conflict of Interest

The authors declare that the research was conducted in the absence of any commercial or financial relationships that could be construed as a potential conflict of interest.
