# Supplementary material for: High Expression of IL-36γ in Influenza Patients Regulates Interferon Signaling Pathway and Causes Programmed Cell Death During Influenza Virus Infection
Source: Front Immunol. 2020 Oct 22;11:552606. doi: 10.3389/fimmu.2020.552606 (PMC7642405; doi:10.3389/fimmu.2020.552606)
Supplement: Supplementary file 1 [file DataSheet_1.pdf]

Supplementary Material

Supplemental Table 1

Demographic and clinical data of influenza-induced ARDS patients

Characteristic	Flu-induced ARDS patients (n=20)
**Age (mean±SD, year)**	53.2±14.5
**Male (n, %)**	13/20
**Flu virus strains**	
Influenza type A	16/20
Influenza type B	4/20
**Comorbidities**	
Pneumonia disease	20/20
COPD	0/20
Asthma	0/20
Bronchiectasis	1/20
Diabetes	5/20
Kidney disease	10/20
Skin disease	0/20
**Clinical features**	
Fever	20/20
Cough	20/20
Sputum	12/20
Hemoptysis	1/20
Consciousness Disorders	1/20
**Laboratory tests**	
Total leukocytes ($\times 10^9$/L)	9.8±5.5
Neutrophil ($\times 10^9$/L)	8.6±5.2
Lymphocytes ($\times 10^9$/L)	0.8±0.4
Hemoglobin (g/L)	122±24.8
Platelet ($\times 10^9$/L)	178.4±88.6
Creatine kinase (U/L)	519.1±998.5
LDH (U/L)	611.1±313.9
**PaO_2/FiO_2 grade (mm Hg)**	
≥200 to <300	4/20
≥100 to <200	10/20
<100	6/20
**ICU admission**	20/20
**NAI treatment**	20/20
**Use of invasive ventilation**	16/20
**ECMO**	10/20

Note: ECMO = Extracorporeal membrane oxygenation

Supplemental Table 2

The primer used in the RT-PCR

Primer	Sequence (5'-3')	Use For
**IL-36A**	F: GACCAGACGCTCATAGCAG<br>R: CTTTAGCACACATCAGGCAG	Q-PCR for detecting human *IL-36A* gene
**IL-36B**	F: ACAAAAAGCCTTTCTGTTCTATCAT<br>R: CCATGTTGGATTTACTTCTCAGACT	Q-PCR for detecting human *IL-36B* gene
**IL-36G**	F: GGTCAGAACCTTGTGGCAGT<br>R: CAAGAGCCTCTGGATACTTGC	Q-PCR for detecting human *IL-36G* gene
**IL-18**	F: AAGACTCTTGCGTCAACTTCAAGGA<br>R: AGTCGGCCAAAGTTGTCTGATTC	Q-PCR for detecting human *IL-18* gene
**G-CSF**	F: TCCCCATCCCATGTATTTATCT<br>R: AACTCAGAAATGCAGGGAAGGA	Q-PCR for detecting human *G-CSF* gene
**CCL20**	F: TCTGTGTGCGCAAATCCAA<br>R: CCATTCCAGAAAAGCCACAG	Q-PCR for detecting human *CCL20* gene
**CXCL3**	F: CGCCCAAACCGAAGTCAT<br>R: GTGCTCCCCTTGTTCAGTATC	Q-PCR for detecting human *CXCL3* gene
**IL-6**	F: TAATGGGCATTCCTTCTTCT<br>R: TGTCCTAACGCTCATACTTTT	Q-PCR for detecting human *IL-6* gene
**CXCL8**	F: AGGGTTGCCAGATGCAATAC<br>R: GCAAACCCATTCAATTCCTG	Q-PCR for detecting human *CXCL8* gene
**IL-12**	F: TGGAGTGCCAGGAGGACAGT<br>R: GTTGATGTCCCTGATGAAGAAGC	Q-PCR for detecting human *IL-12* gene
**IFNG**	F: GCAGAGCCAAATTGTCTCCT<br>R: ATGCTCTTCGACCTCGAAAC	Q-PCR for detecting human *IFNG* gene
**IL-36R**	F: GCTGGAGTGTCCACAGCATA<br>R: GCGATAAGCCCTCCTATCAA	Q-PCR for detecting human *IL-36R* gene
**IL-1RAcp**	F: AAGACAGCTGTTTCAATTCC<br>R: TTGAAATTAAGGCAATGAGG	Q-PCR for detecting human *IL-1Acp* gene
**COX-2**	F: GTCCTGGCGCTCAGCCATACAG<br>R: TCCTGTCCGGGTACAATCGCAC	Q-PCR for detecting human *COX-2* gene
**OAS**	F: AGAAGGCAGCTCACGAAACC<br>R: CCACCACCCAAGTTTCCTGTA	Q-PCR for detecting human *OSA* gene
**PKR**	F: AGAGTAACCGTTGGTGACATAACCT<br>R: GCAGCCTCTGCAGCTCTATGTT	Q-PCR for detecting human *PKR* gene
**Mx1**	F: GAGAGGAAACTGTAGGGGA<br>R: GGAAACATCTGTGAAAGCAA	Q-PCR for detecting human *Mx1* gene
**IAV-NP**	F: TCAGTGATTATGATGGACGACTA<br>R: GCACTGGGATGCTCTTCTAGGTA	Q-PCR for detecting IAV-NP
**NP-mRNA**	Oligo DT	For reverse transcription
**NP-vRNA**	AGCAAAAGCAGG	For reverse transcription

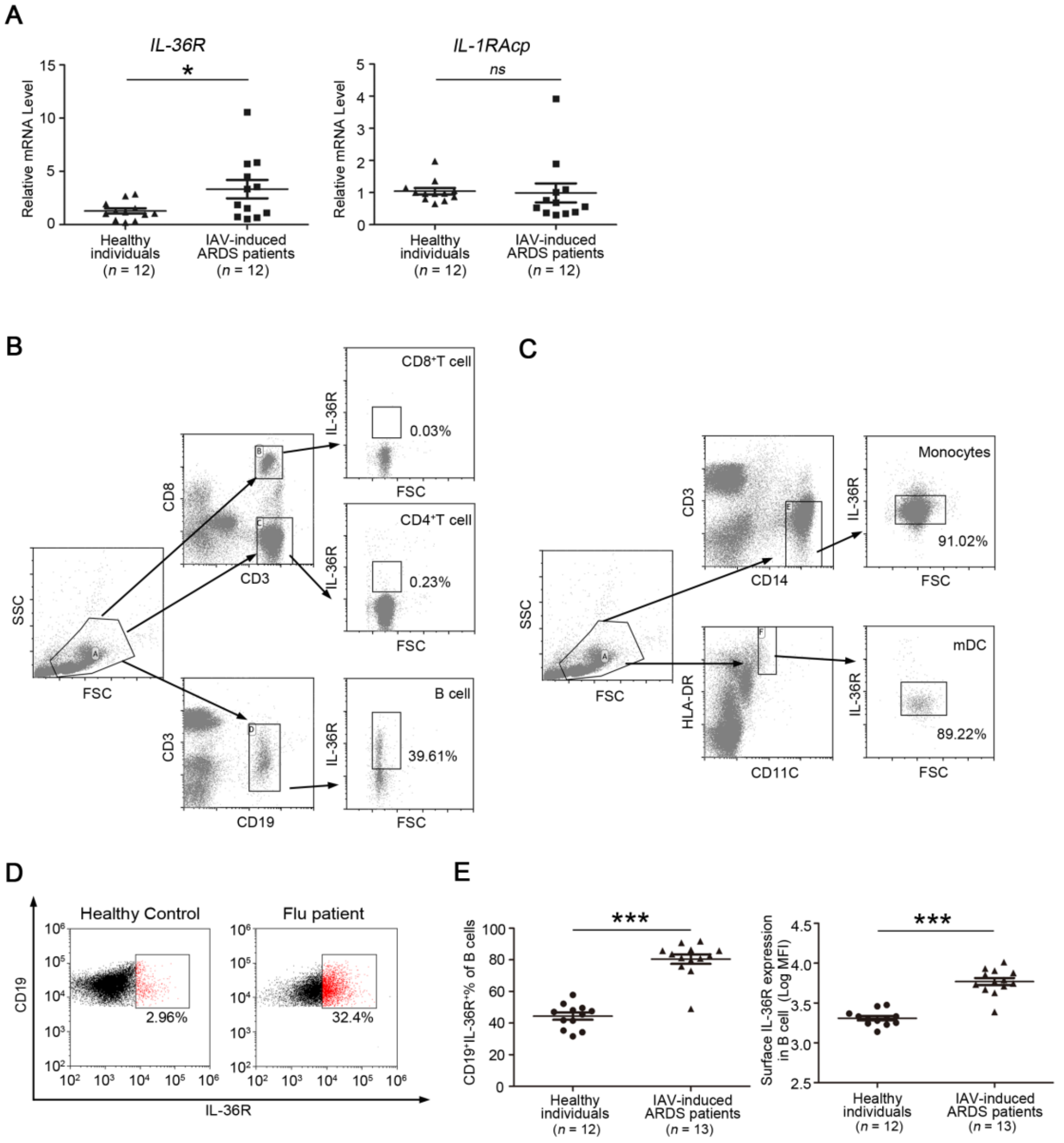

Supplemental Figure 1.

(A) The relative mRNA expression levels of *IL-36R* and *IL-1RAcp* in PBMCs of Influenza A virus (IAV)-induced ARDS patients (*n* = 12) and healthy individuals (*n* = 12) were detected by RT-PCR. **(B, C)** The gating strategy and representative flow plots. IL-36R expression on peripheral blood mononuclear cells (PBMC) isolated from whole bloods were detected. **(D)** Representative flow cytometry dot plots of $CD19^{+}IL\text{-}36R^{+}$ lymphocytes gated from $CD19^{+}$ B cells in healthy control and IAV-induced ARDS patient. **(E)** $IL\text{-}36R^{+}$ B cells in total B cells and expression levels of IL-36R on B cells from IAV-induced ARDS patients (*n* = 13) and healthy individuals (*n* = 12) were detected using flow cytometry. Results are presented as scatter plots with median of the proportion or mean fluorescence intensity (MFI) subtracting corresponding isotypic controls. Statistical significance is determined by Student's t test. *P < 0.05, ***P < 0.001, *ns*, not significant.

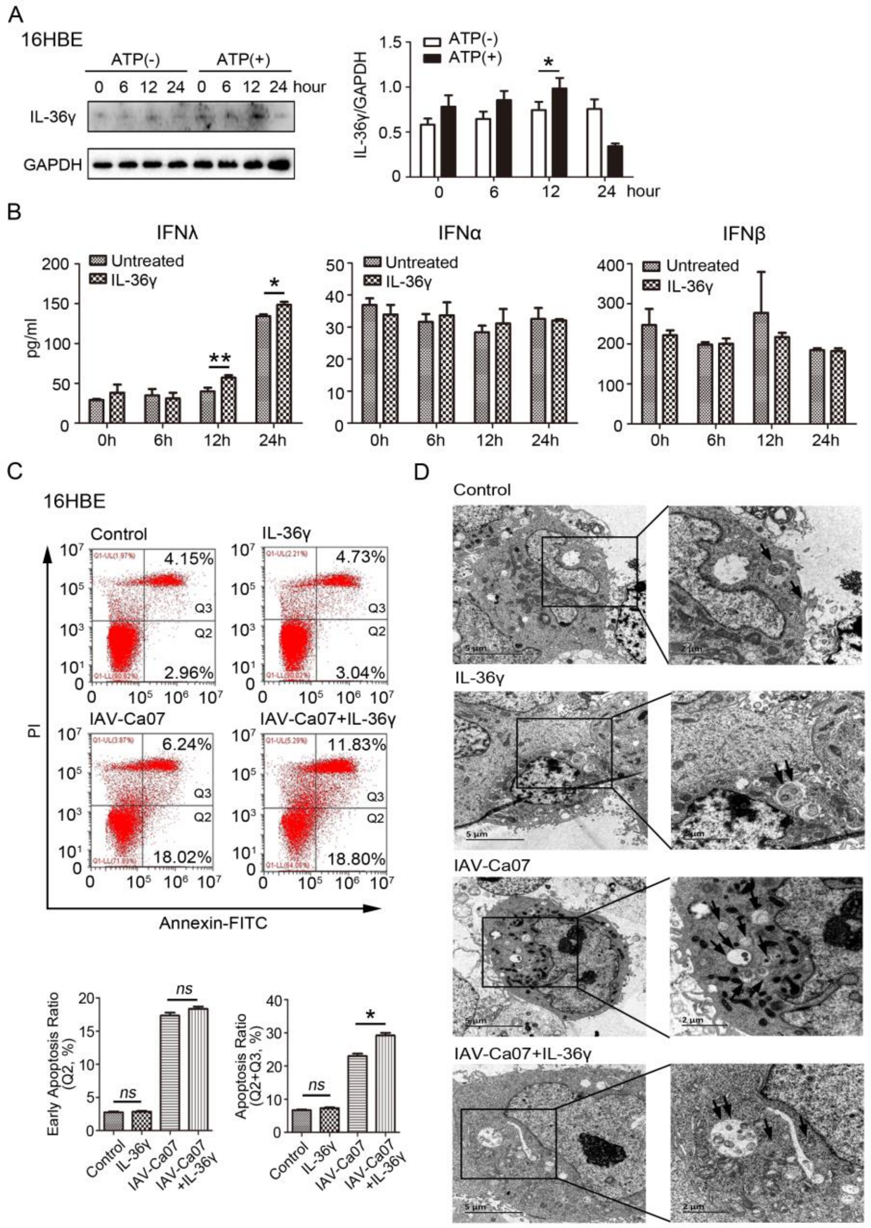

Supplemental Figure 2.

(A) 16HBE cells were infected for the indicated timeframes with IAV (MOI = 1), after this, treated with or without ATP (1 mM, 2 h), intracellular protein level of IL-36γ was examined and analyzed by Western Blotting and Image J. **(B)** 16HBE cells were treated with IL-36γ (100 ng/ml), or left untreated 24 h prior to infection with IAV-Ca07 (MOI = 1). Levels of IFN-λ1, IFN-α and IFN-β in supernatant medium were measured by ELISA in indicated timeframes. **(C)** Apoptotic 16HBE cells were determined by flow cytometry after infected with IAV-Ca07 (MOI = 1) or joined IL-36γ (100 ng/ml) together for 24 h. The early apoptosis ratio and total apoptosis ratio (early apoptosis and late apoptosis) was quantified by Flow J software. The data shown were the mean ± SD of three independent experiments. **(D)** 16HBE cells were infected with or without IAV-Ca07 and treatment with or without IL-36γ for 12 h. Cells were then collected for TEM analysis of the autophagic structures. Representative autophagosomes or autolysosomes (black arrows) are highlighted in the enlarged images (right). Statistical significance is determined by Student's t test. *P < 0.05, **P < 0.01, *ns*, not significant.

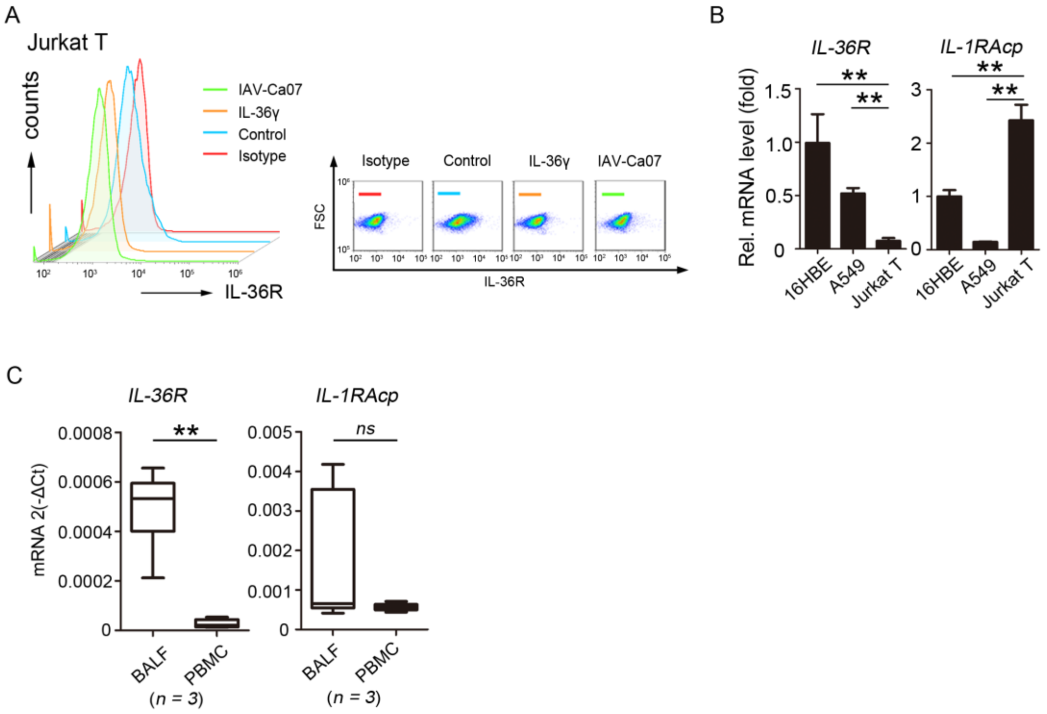

Supplemental Figure 3.

(A) Flow cytometry to reveal IL-36R expression on the surface of Jurkat T cells after treated with IL-36γ (100 ng/ml) or infected with IAV (MOI = 1) for 24 h. **(B)** Relative mRNA expression levels of *IL-36R* and *IL-1RAcP* in A549,16HBE as determined by RT-PCR, and Jurkat T cells served as a control. **(C)** mRNA levels of *IL-36R* and *IL-1RAcp* in human BALF and in PBMCs from influenza-induced ARDS patients (*n* = 3) were detected using RT-PCR. Statistical significance is determined by Student's t test. **P < 0.01, *ns*, not significant.

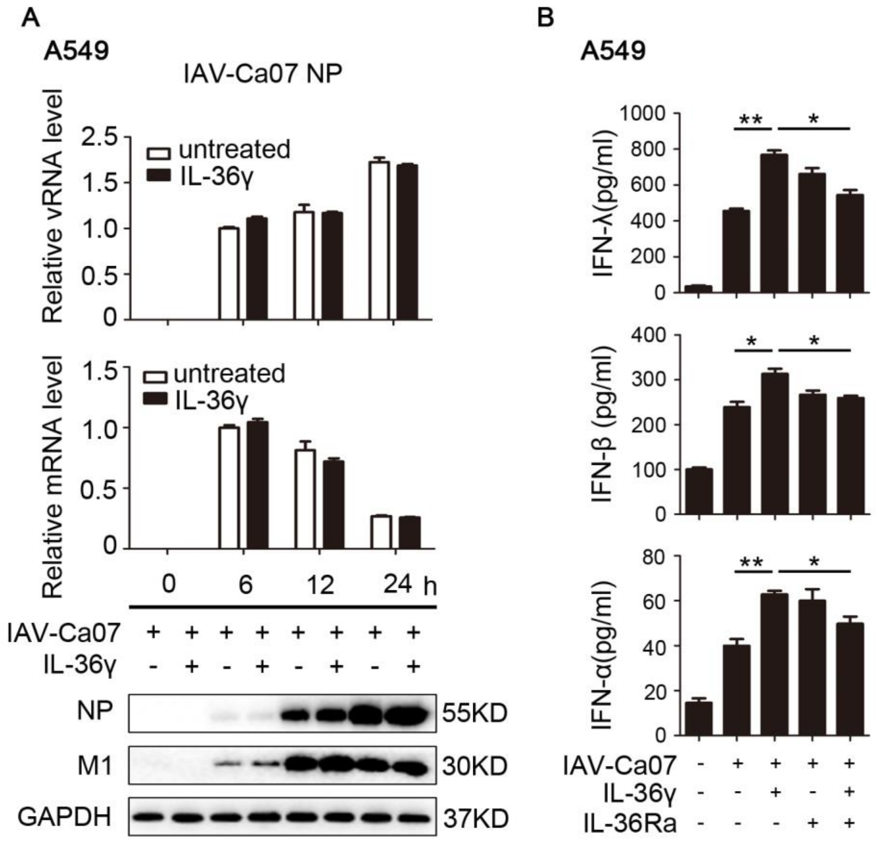

Supplemental Figure 4.

(A) A549 cells were pre-treated with recombinant IL-36γ (100 ng/ml) for 12 h before IAV-Ca07 infection (MOI = 1). Cells were harvested for the indicated timeframes. Total RNA was isolated for detection of NP-specific mRNA and vRNA, and total protein was extracted for western blotting to detection of IAV-NP and M1. **(B)** A549 cells were pre-treated with IL-36γ (100 ng/ml) and (or) IL-36Ra (100 ng/ml) prior to IAV-Ca07 challenge. After 24 h, ELISA was performed to measure the IFN-α, IFN-β and IFN-λ1 concentrations in the culture supernatant. Statistical significance is determined by Student's t test. *$P < 0.05$, **$P < 0.01$.
